# Caregiver’s cognitive traits are associated with pup fitness in a cooperatively breeding mammal

**DOI:** 10.1038/s41598-023-44950-6

**Published:** 2023-10-18

**Authors:** C. Shelafoe, F. J. Thompson, F. Mwanguhya, S. Kyabulima, R. Businge, K. Mwesige, J. L. Sanderson, M. A. Cant, H. H. Marshall, E. I. K. Vitikainen

**Affiliations:** 1 Centre for Research in Ecology, Evolution, and Behaviour, University of Roehampton, London, SW15 5PJ UK; 2 Centre for Ecology and Conservation, University of Exeter, Penryn, Cornwall TR10 9FE UK; 3 Banded Mongoose Research Project, Queen Elizabeth National Park, PO box 66, Lake Katwe, Kasese District Uganda; 4 Centre for Conservation Science, Royal Society for the Protection of Birds, Cambridge, UK; 5 Organismal and Evolutionary Biology, University of Helsinki, PO Box 65, 00014 Helsinki, Finland

**Keywords:** Zoology, Animal behaviour

## Abstract

Studies across diverse taxa have revealed the importance of early life environment and parenting on characteristics later in life. While some have shown how early life experiences can impact cognitive abilities, very few have turned this around and looked at how the cognitive skills of parents or other carers during early life affect the fitness of young. In this study, we investigate how the characteristics of carers may affect proxies of fitness of pups in the cooperatively breeding banded mongoose (*Mungos mungo*). We gave adult mongooses a spatial memory test and compared the results to the success of the pups those individuals cared for. Our results show a tradeoff between speed and accuracy in the spatial memory task, with those individuals which were faster to move between cups in the test arena making more erroneous re-visits to cups that they had already checked for food. Furthermore, the accuracy of their carer predicted future survival, but not weight gain of the pups and the effect was contrary to expected, with pups that were cared for by less accurate individuals being more likely to survive to adulthood. Our research also provides evidence that while younger carers were less accurate during the test, the age of the carer did not have an impact on the chance of raising young that live to sexual maturity. Our findings suggest that banded mongoose carers’ cognitive traits have fitness consequences for the young they care for, affecting the chance that these young live to maturity.

It is known that early life conditions can have a strong impact on an individual’s social and reproductive characteristics, such as future parental care^1^, fitness^2,3^, and dominance^4^. Several studies provide evidence that the long-term reproductive success of young is affected by the parental care that they receive^2,5^. For example, western gulls *(Larus occidentalis)* have been shown to produce more successful young per clutch as they age, which has been attributed to greater experience and maturity^6^.

Several studies have shown that early life conditions also affect cognitive abilities^7,8^ and tool use^9^. For example, hand-reared individuals versus mother-reared showed a difference in behaviour and cognitive abilities, such as social learning and tool use in capuchin monkeys (*Cebus apella*)^9^. Additionally, maternal care during early life influences hippocampal growth, spatial memory, and learning in rats (*Rattus sp.*)^10,11^. Since early life conditions and care received by young can affect many characteristics, including cognition, those characteristics could affect a parent's ability to provide that care. Therefore, we wanted to look more into how cognitive traits of a carer would affect the young they rear, specifically from a species that receives care from a nonparent.

Spatial memory is the ability to remember and track one's location in space^12^. This type of cognition is critical for foraging patterns and foraging success^13^. Since this type of cognition influences foraging, we hypothesized that it would also influence one's ability to rear young, especially where this involves providing young with resources collected from the environment. Spatial cognitive abilities are at similar levels in individuals from different species with analogous foraging strategies^14^. Furthermore, food scouts have better spatial memory than food collectors in social redwood ants (*Formica rufa*)^14^. This difference in cognitive abilities fits since the scouts must find the food and find their way back, whereas collectors follow the leader. This kind of differences in ability may link to individual preferences or division of labour, but may also be linked to the individual's fitness, for example, through differences in foraging efficiency. Even in captivity, western lowland gorillas (*Gorilla gorilla gorilla)* with better spatial cognition were more successful and efficient when foraging for food hidden in their enclosure^15^.

When measuring cognitive abilities, such as spatial memory, it is essential to consider the relationship between an individual’s accuracy in decision-making and the speed at which those decisions are made. A negative one-to-one relationship is found between speed and accuracy in humans^16^. Similar results indicated that the slower the response, the better the accuracy in buff-tailed bumblebees (*Bombus terrestris)*^17^ and honeybees (*Apis sp.*)^18^. In zebrafish (*Danio rerio),* some individuals consistently made quick inaccurate moves, while others made slow moves with few errors^19^. As a result, measuring speed and accuracy together gives a better measure of cognitive abilities than measuring either speed or accuracy on their own^20^. With various papers showing a tradeoff between speed and accuracy^21,22^, we concluded that measuring both would be the best way to account for this tradeoff. Here, we combine data from a long-term field study of cooperatively breeding banded mongooses (*Mungos mungo)*^23^ with arena tests of adult carers, to investigate whether cognitive traits of the carers associate with fitness of the pups they care for. The arena test was based on the “free choice” type cognitive hole board design^24^, which have been frequently used in assessing spatial learning and memory across species, especially in rodents and humans. In the arena test food is hidden in several locations and most effective foraging strategy involves using spatial memory, to avoid re-checking locations.

We asked, does having better spatial memory allow an individual to raise more successful young? In the banded mongoose, after the first month of a pup's life, it pairs up in a one-to-one relationship with an adult called an escort. The escort grooms, feeds, protects, carries, and plays with the pup during the escorting period, and the relationship is vital to the pup’s survival^25^. The escorting relationship makes banded mongooses an unusually suitable model species for studying the effect of early-life conditions on success because the pup is not directly related to the escort, which allows for the isolation of the nurture portion of early-life conditions^26,27^. Previous work has highlighted the effects that the caring relationship has on both short and long term fitness of pups^26,27^. Importantly, escort relationship has a long lasting effect also on pup foraging behaviour, with pups adopting the foraging style and even niche from their carers, rather than from their parents^28^. Banded mongooses forage on invertebrates which are discretely distributed across the environment, often requiring mongooses to dig or search through dense undergrowth to find them. Spatial memory is, therefore, likely to be a key cognitive skill to allow mongooses to forage efficiently by minimising them returning to locations they have already searched. We used this escort-pup system to test the hypothesis that carer spatial memory influences the success of the young being reared. We predict that the mongooses that make more accurate or faster decisions in tests of spatial memory will rear young that are in better condition at independence and more likely to live to sexual maturity.

## Methods

### Study site and field data collection

We used a combination of data collected from videos and data from a long-term observational study of a wild banded mongoose population at Queen Elizabeth National Park in Uganda (0°12′S, 29°54′E). The cognitive experiments were run between January 2014 and March 2015, and during this time the standing population size was around 250 individuals making up 12 social groups. All individuals in the population have been tagged with either plastic collars, a fur shave pattern, or a fur dye pattern, allowing them to be individually identified by sight. In order to maintain the markings, all individuals are trapped every 3–6 months using Tomahawk Live Traps^29,30^. In short, small pieces of fish or dog food were used to bait the animals inside. Once baited, the traps were placed in a well-shaded and protected area at dawn. The traps were checked at least every two hours until most group members were captured, and all traps were closed at 1600 h^29^. Individuals were anesthetized using isoflurane inhalation (for procedure details, see Jordan et al.^30^). There have been no detectable adverse effects of the trapping procedures in 22 years/2602 trapped individuals, and the process has been approved by the Uganda Wildlife Authority (UWA) and the Uganda National Council for Science and Technology (UNCST).

A 26-g radio collar (< 2% of body mass, Sirtrack Ltd.) with a 20-cm whip antenna is fitted to one or two adults in each pack, which allows the pack to be located. Each pack is visited every 1–3 days to record demographic and life history data, i.e. births and presence of individuals in the social groups. This allows for accurate data on age and identity of individuals^23^. During the experiment, six packs that are habituated to humans could be observed at 5-m and visited every few days to collect behavioural and life history data. Habituated individuals have body mass (g) measured without trapping by coaxing the individual onto a scale with a small milk reward. In addition, each pack is visited once or twice daily to record escorting behaviour during the escorting period. An individual is classified as escorting a pup when it is within 0.3-m of the pup for more than half of a 20-min observational period^28^. For more information on the study site and protocols, see^31^ and^29^.

### Spatial memory trials

A spatial memory test was conducted to assess individual speed and efficiency in a foraging task. The arena test was set up to quantify (i) the individual’s ability to retrieve food from separate locations within the study arena, without revisiting locations it has already checked, and (ii) the speed with which it performs this task. We assumed that individuals with better spatial memory would be more accurate, i.e. do fewer re-visits, and that performance speed may be (inversely) linked with accuracy^32^. In total, there were 119 individuals tested between January 2014 and March 2015 in Uganda during routine trapping events. The mongooses that participated in this test were selected from the wild habituated population routinely trapped for markings maintenance. The individuals are kept in cages until they are ready to be released after having their markings maintained. The animal’s performance in an arena test was assessed before the animal was anaesthetized, to avoid any effects of anesthetics on test performance. Care was taken to keep the mongooses’ stress levels down during the test. This was done by covering the cage in a black bag, being quick with tasks, moving around them quietly and keeping them in quiet area whilst they were in cages.

**Figure 1:**
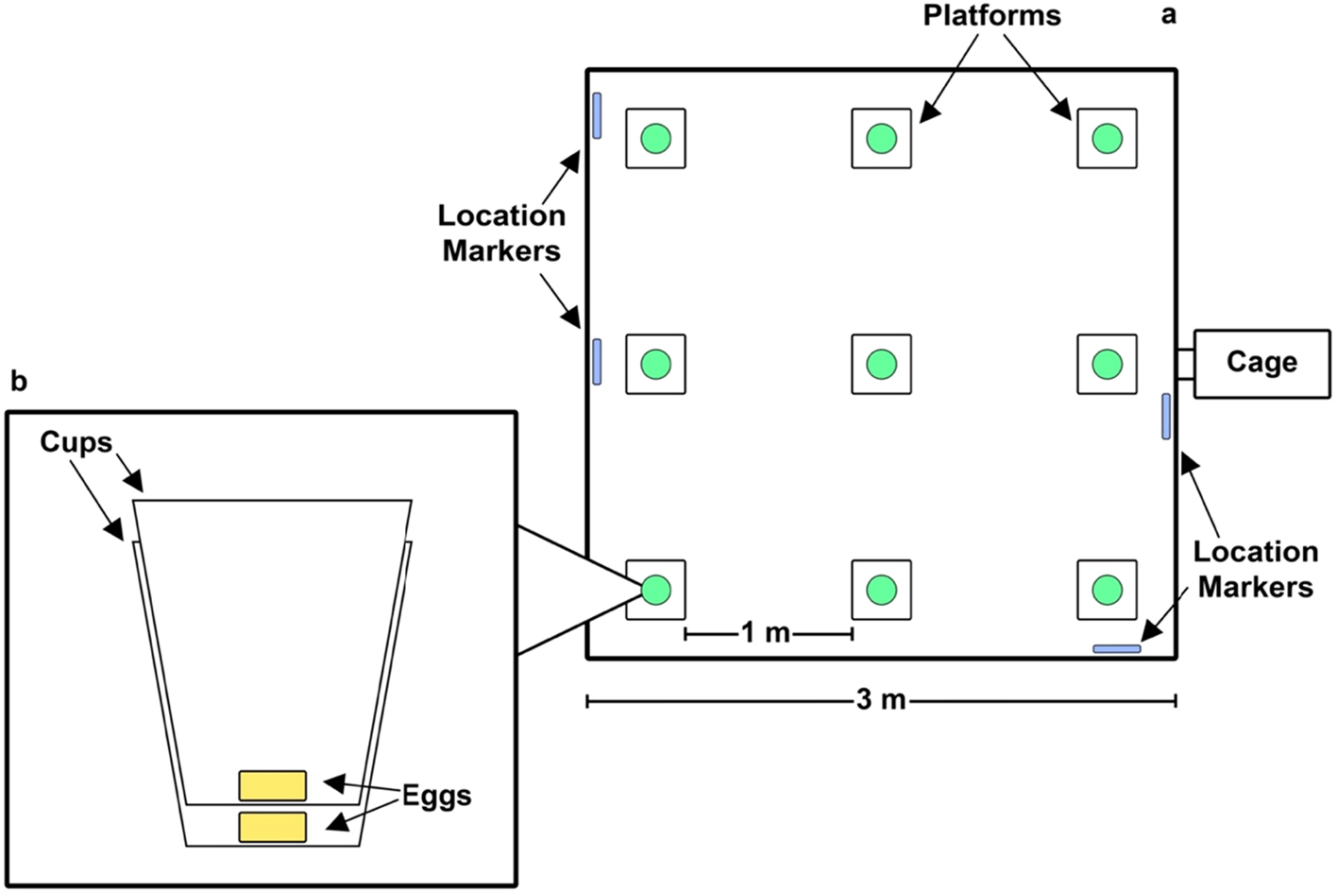
(**a**) Top-down view—The enclosure diagram with the test arena setup for the videos. Green circles represent the cups, and unique location markers were place on the walls to allow for spatial orientation inside the arena. (**b**) Side view—A diagram of the stacked cups and where the egg pieces are in the cup.

**Figure 2:**
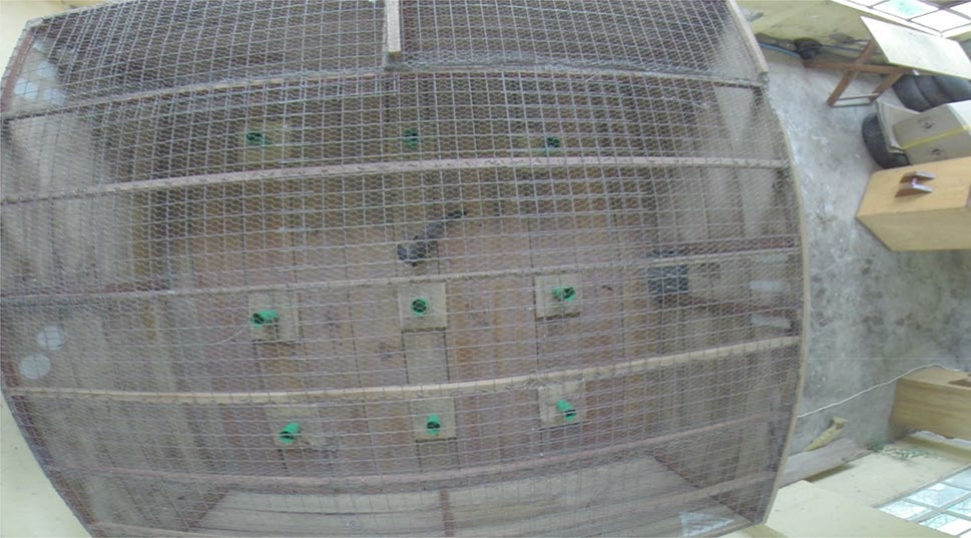
Screenshot from one of the trial videos. The 3 m by 3 m testing arena with the green cups containing pieces of egg. The cups are spaced 1 m apart. Unique location markers can be seen on the arena walls in the top right and bottom left of the picture.

Each test took 10 min and was recorded on a GoPro Hero 4 attached to the ceiling above the test arena and remotely operated from outside the room containing the arena. The test arena (Figs. 1a and 2) was a 3-m × 3-m wooden enclosure with 1-m-high walls and a wire mesh top, containing nine platforms spaced 1 m apart. Each platform (Fig. 1b) consisted of a 30 cm × 30 cm wooden board with a plastic cup nailed to it and a second cup pushed firmly inside the first. Each cup contained a piece of cooked egg. The piece of egg in the second (top) cup could be accessed by the mongoose. The egg in the first (bottom) cup was not accessible but could be smelled by the mongooses through holes drilled in the cup sides. The rationale for this bottom, inaccessible piece of egg was to keep the platform smelling of egg after the egg in the top cup has been eaten, ensuring mongooses used spatial memory rather than olfactory cues to assess which platforms they had already visited. Test arena was also thoroughly cleaned between individuals, to ensure that mongooses were not aided nor distracted by possible scent marks from other individuals.

The arena contained four unique location markers on its walls so the mongooses could orient themselves. The cage the mongooses were kept in after trapping fit inside an opening in the enclosure wall and a cord attached to the cage door could be hooked up to keep the cage door open. At the start of each trial the cage door was opened, allowing the mongooses access to the test arena, after which we left the room. Each trial lasted for 10 min. 5 individuals did not leave the cage during the 10 min, and their trials were excluded from the analysis. The total number of trials run was 219. The mean number of trials an individual did was 1.8.

The videos were subsequently scored to extract data on each mongoose’s performance in the trials. We measured performance in two ways:

1. The average time between one cup visit and the next, referred to as “time” hereafter. We defined a cup visit as when a mongoose put its head inside a cup.
2. How many mistakes were made, referred to as “errors” hereafter. Errors were defined as an individual returning to a cup after they had already eaten the egg it contained.

All data collection and experimental procedures, including anaesthesia for maintenance of individual identification marks, were performed in accordance with the ASAB guidelines for the treatment for animals in research, and under permissions from the Uganda Wildlife Authority (UWA) and Uganda National Council for Science and Technology (UNCST), and approved by the University of Exeter Ethics Committee. This research also complied with the Animal Research: Reporting of In Vivo Experiments (ARRIVE) guidelines^33^.

### Data analysis

We analyzed our data using R statistical software^34^. Generalized linear mixed models were fitted using the lme4 package in R^35^, as described below. Model fit was confirmed by visual investigation of the residuals, which were homogenous and normally distributed for all models, confirming that model assumptions were met. We used MuMIn^36^ to calculate the marginal trigamma value as well as the conditional r^2^, taking into the variance explained by random terms included in the models, and these are reported below. The Poisson models did not include an offset, because trial time was the limiting factor and it was the same (10 min) for all individuals that participated in the experiment.

### Speed-accuracy tradeoff model

We tested whether there was a tradeoff between the time an individual spent moving between the cups (“time”) and the number of errors they made (repeat visits to a cup they had already explored). We fitted a Poisson model using log link function with the number of errors as the response variable and time as the fixed effect. To control for repeated measures, we included individual identity and social group as random intercepts.

### Spatial memory trial performance models

We constructed two separate models to test for effects on speed and accuracy in the spatial memory trials, as the speed-accuracy model indicated the two were heavily correlated (see Results). Speed and accuracy data were both counts so we used a log link function and a Poisson error structure in both models. Both models included sex and age as fixed effects and as in the speed-accuracy model above, individual identity and social group were included as random intercepts to control for repeated measures. The first model had the number of errors as the response variable, and the second model had time between cups as the response variable. Both models had a lower correlation between fixed effects than the threshold coefficient previously shown to cause issues in effect estimates (max r = 0.26)^37^.

### Pup fitness models

We constructed four models to test whether the number of errors that the escort made or the time they spent doing the cognitive task was connected to two proxies of fitness in the pups that the individual had escorted: their weight at independence and whether the pup survived to sexual maturity (1 year of age).

The weight models had pup weight (grams) at independence as the response variable, a Poisson error structure, and a log link function. The fixed effects variables in both weight models were the sex of the pup, the age (days) at the weight measurement, the age (days) of their escort, and cumulative rainfall (mm) during the escorting period. Escort age was included in both weight models to control for potential effects it could have, for example, through escort experience or varying escorting effort with age. The error-weight model also had the number of errors as a fixed effect, while the time-weight model included the average time spent between cup visits. The random effects were escort identity and social group to control for repeated measures from each of these. Both models had a lower correlation between fixed effects than the threshold coefficient previously shown to cause issues in effect estimates (max r = 0.25)^37^.

The two survival models had pup survival to one year (0/1) as the response variable, a logit link function, and a binomial error structure. Escort age was included as fixed effects as well as pup sex to account for potential sex-specific effects on survival, see Vitikainen et al.^38^. The error-survival model had the number of errors as a fixed effect, and the time-survival model had a fixed effect of time spent between cup visits. The random effects for both survival models were escort identity and social group to control for repeated measures. Both models had a lower correlation between fixed effects than the threshold coefficient previously shown to cause issues in effect estimates (max r = 0.27)^37^.

## Results

### Speed-accuracy tradeoff model

**Figure 3:**
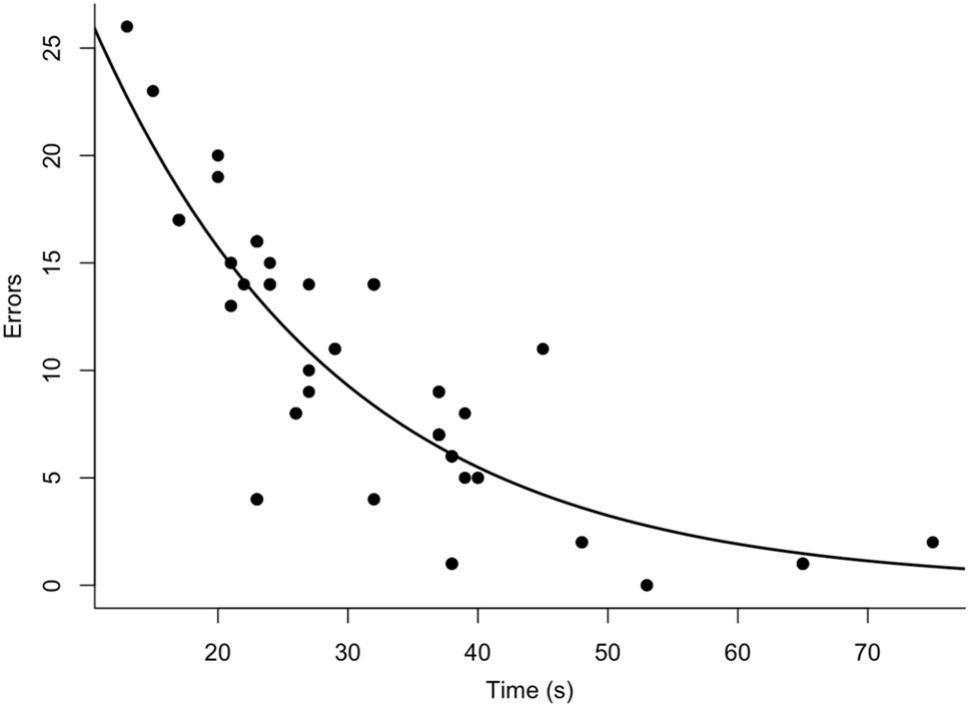
The relationship between errors that individuals made in the trials (number of repeat visits to cups containing egg) and the average time they spent between cup visits. The line shows the relationship predicted by our speed-accuracy tradeoff model and points are raw data.

Individuals that spent more time between cup visits made less errors, i.e. made fewer repeat visits (Intercept ± S.E. = 3.81 ± 0.12, z = 32.78, *p* =  < 2.00 × 10^–16^; Effect ± S.E. = − 0.05 ± 4.13 × 10^–3^, z = − 12.73, *p* =  < 2.00 × 10^–16^; Fig. 3). The marginal/conditional trigamma r^2^ value for the model was 0.93/0.97.

### Spatial memory trial performance models

**Figure 4:**
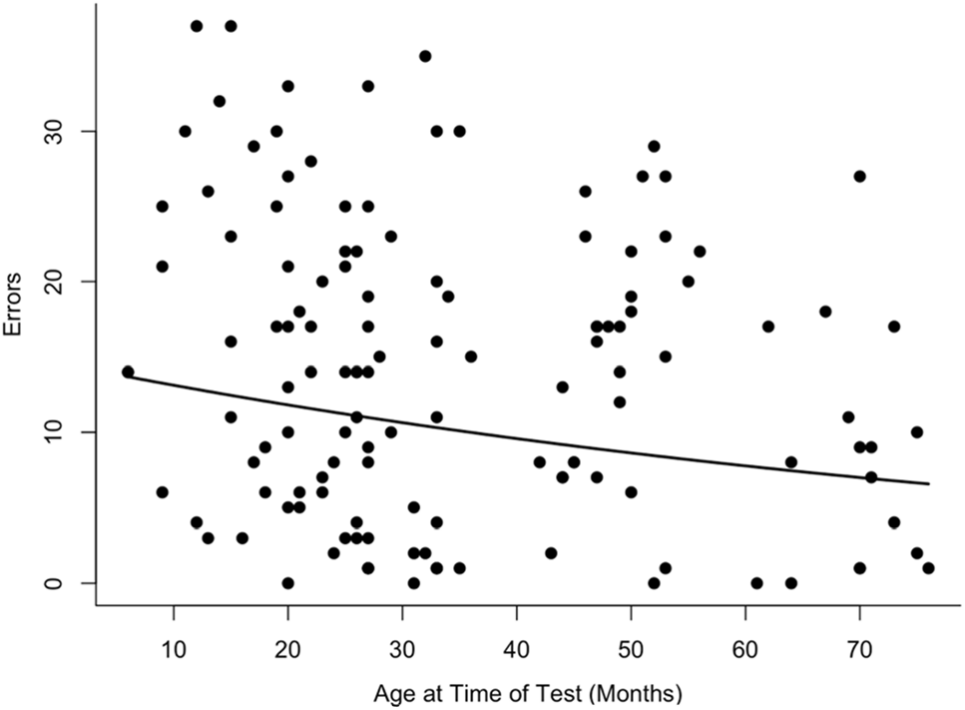
The relationship between age at the time of the test and the number of errors (repeat cup visits) the individual made. The line shows the predicted relationship based on the model in Table 1 and points are raw data.

The median number of errors an individual made was 8.5 (IQR = 4–14, n = 92). Younger escorts made more mistakes (Fig. 4). Sex did not predict the number of errors made. The median time between cup visits was 32 s (IQR = 23.75–38, n = 92). Age and sex had no impact on the time spent between cup visits. All results from the spatial memory trial models are reported in Table 1.

**Table 1:** Generalized linear mixed model results for the cognitive test performance models.

Response variable	Explanatory variables	Estimate	Standard error	Z value	P value
Errors (repeat cups)	Intercept	2.54	0.27		
	Sex (Male)^a^	0.14	0.18	0.79	0.43
	**Age (Days)**	**− 0.01**	**4.75 × 10**^**–3**^	**− 2.22**	**0.03**
Time between cups	Intercept	3.21	0.16		
	Sex (Male)^a^	− 0.13	0.12	− 1.14	0.26
	Age (Days)	3.89 × 10^–3^	3.01 × 10^–3^	1.29	0.20

^a^Reference category = Female.

Significant values are in bold. The marginal/conditional trigamma r^2^ value for the errors model was 0.04 /0.91 and for the time model 0.02/ 0.90.

### Pup fitness models

The mean ± standard error of pup weight at nutritional independence was 578.8 ± 141 g (n = 106). The older the pup, the heavier they were (mean ± standard error age at weighing = 105 ± 27 days). Neither escort performance in the cognitive test (errors, time between tests) nor its age, the sex of the pup, nor rainfall predicted pup weight at independence. All results of pup weight models are reported in Table 2.

**Table 2:** Generalized linear mixed model results for the time and error pup weight models.

Model	Explanatory variables	Estimate	Standard error	Z value	P value
Error	Intercept	5.48	0.17		
	errors	3.51 × 10^–3^	3.40 × 10^–3^	1.03	0.30
	Sex (Male)^a^	0.07	0.04	1.82	0.07
	**Age for weight at independence (Days)**	**6.96 × 10**^**–3**^	**1.34 × 10**^**–3**^	**5.18**	**2.17 × 10**^**–7**^
	Rainfall (mm)	− 2.91 × 10^–4^	3.10 × 10^–4^	− 0.94	0.35
	Escort age (Days)	1.96 × 10^–5^	5.17 × 10^–5^	0.38	0.70
Time	Intercept	5.51	0.17		
	Time	3.66 × 10^–4^	1.64 × 10^–3^	0.22	0.82
	Sex (Male)^a^	0.06	0.04	1.70	0.09
	**Age for weight at independence (Days)**	**7.03 × 10**^**–3**^	**1.34 × 10**^**–3**^	**5.23**	**1.69 × 10**^**–7**^
	Rainfall (mm)	− 2.73 × 10^–4^	3.15 × 10^–4^	− 0.87	0.39
	Escort age (Days)	5.81 × 10^–6^	5.23 × 10^–5^	0.11	0.91

^a^Reference category = Female.

Significant values are in bold. The marginal/conditional trigamma r^2^ value for the error-weight model was 0.17/0.97 and for the time-weight model 0.16/0.97.

**Figure 5:**
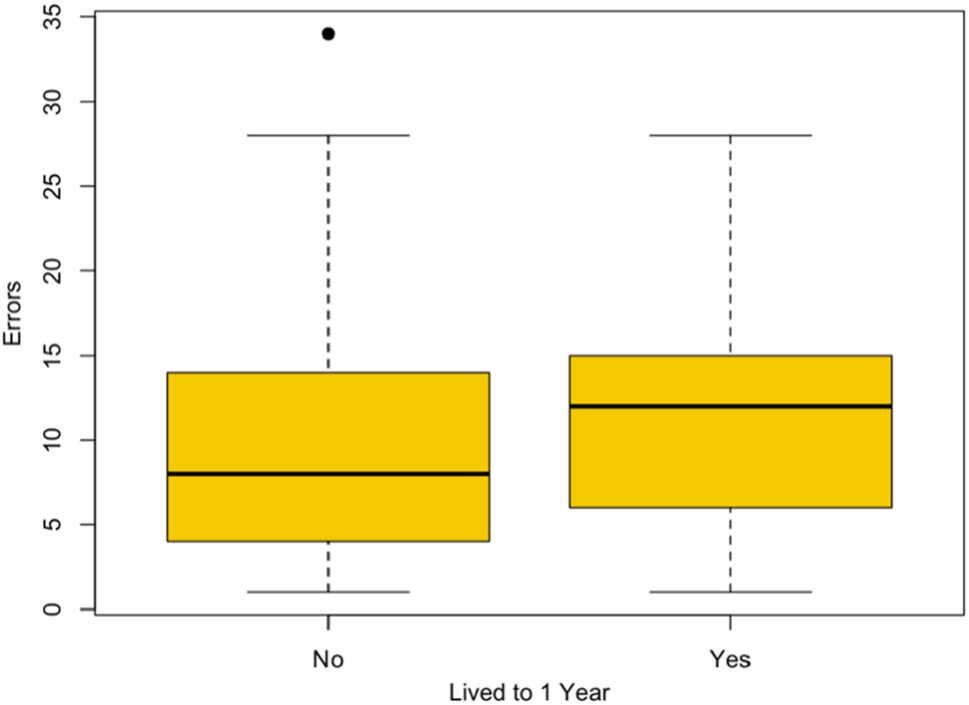
The number of errors made by escorts of pups that did or did not live to sexual maturity (one year). The yellow box represents the interquartile range. The solid black line is the median, and the top and bottom of the dashed line show the minimum and maximum values. The black dot represents an outlier.

Pups escorted by individuals that made more errors in the cognitive test were more likely to live to sexual maturity (Fig. 5). Time spent between cup visits, the age of the escort, and sex did not predict pup survival. The pup survival model results are reported in Table 3.

**Table 3:** Generalized linear mixed model results for the pup survival models, with the number of errors (repeat cup visits) and time spent between cups as predictors.

Model	Explanatory variables	Estimate	Standard error	Z value	*P* value
Error	Intercept	− 0.13	1.04		
	**Errors**	**0.09**	**0.04**	**2.13**	**0.03**
	Sex (Male)^a^	0.19	0.48	0.42	0.68
	Escort age (Days)	4.48 × 10^–5^	5.99 × 10^–2^	0.08	0.94
Time	Intercept	1.88	0.96		
	Time	− 0.03	0.02	-1.63	0.10
	Sex (Male)^a^	0.09	0.47	0.20	0.84
	Escort age (Days)	− 6.34 × 10^–5^	5.75 × 10^–4^	-0.11	0.91

^a^Reference category = Female.

Significant values are in bold. The marginal/conditional r^2^ value for the error-survival model was 0.07/0.26 for the time-survival model 0.04/0.22.

## Discussion

Our study aimed to test if the spatial memory of adult helpers correlated with the short and long-term fitness of the pups they cared for. To do this, we assessed spatial memory of adults from a habituated population of banded mongooses in Uganda, using an arena test based on a food motivated cognitive hole board design. Many studies have provided evidence that the period of parental care is an important time during the development of young^10,39^. This holds true for banded mongooses as well^40^. Most mammals receive care from their biological parents. However, a unique aspect of banded mongooses’ care is that the pups associate with helpers termed escorts, which typically are not their parents^38^ and whose care has long-lasting effects on their future fitness^27,29^; furthermore, a pup has to initiate a relationship with the escort^41^. Other cooperatively breeding mammal species have shown that successful foraging makes for a better provisioner^42,43^, so our reasoning was that individual performance in a foraging task could be linked to the fitness of the pups they cared for.

In this study, our data shows a tradeoff in the speed and accuracy of banded mongooses in spatial memory trials, with individuals that were faster to move between cups making more mistakes, i.e. visiting locations they had already checked for food. The result supports the hypothesis of a tradeoff, especially regarding spatial memory^44^. Surprisingly, our results show that for escorts, making more mistakes during the trial was associated with an increased, not decreased, likelihood of caring for young that live to sexual maturity. This finding was opposite to the expected, and we currently do not know how this association arises. It is possible that the errors made by escorts in the arena test correlate with mistakes made during foraging in the wild (and observed by pups), which some studies suggest could act to increase the effectiveness of teaching^45–47^. For example, research on starlings (*Sturnus vulgaris*) shows an individual watching a conspecific make consistent mistakes while foraging learns faster than those watching either consistent, correct decisions or both incorrect and correct ones^45^. However, there was no relationship between escort accuracy and pup weight at independence, which might be expected if watching escorts make errors would teach the pups better foraging skills and/or boost their survival through weight gain. We currently do not know if there is a direct link between escort cognitive skills and pup survival, and this correlation could be driven by other factors. One possible driving factor could be assortative pairing, where healthier and fitter pups pair up with better escorts that also make more errors (repeat visits) in the foraging test. A possible explanation for this correlation could be that the type of spatial memory tested in the trial is not readily used in their everyday foraging and that, instead, it is an advantage for the mongooses to repeatedly check places where food has been found in the past. As there is a trade-off between speed and accuracy, selection for increased speed during foraging could lead to more successful individuals being less accurate; however, despite this strong negative correlation, only the number of errors and not speed was associated with pup fitness. Repeated checking of cups could nevertheless be an indication of activity level or hunger.

In contrast to the analyses of pup survival, there was no relationship between the accuracy or time spent at the task and short-term fitness benefits to the pups, measured as weight at nutritional independence. This supports the idea that the future fitness benefits are not simply a result of individuals that had a higher error rate for example giving more food to the pups, and that effects are mediated by other aspects of the escort-pup relationship than simple weight gain. One could speculate that the correlations with cognitive skills of escort and pup fitness may only be evident in the long term. This could be because the effects take longer than the 2 months of the escorting period to manifest, for example, through continued learning and individual development after nutritional independence. Other research on banded mongooses shows that parental care and early-life conditions affect an individual’s fecundity later in life^27,39,48^. Our results indicate that the carer cognitive traits may have effects on pups that may not manifest in terms of body mass increase in the short term but could still affect whether an individual lives long enough to start reproducing.

Our study has limitations that are important to note. First, although we made sure to exclude the possibility that individuals could use scent trails of cues from other individuals to navigate the test arena, the spatial memory trials could not guarantee that the mongooses were solely using spatial memory, and not smell, to find their way to the eggs. We attempted to compensate for this with a piece of egg between the two cups that the individual could not extract, so the smell of the egg stayed around the cup consistently, but there is no guarantee that this was wholly accounted for it as some cups had still some egg left after the first visit, which could have caused the individual to visit a second time. Second, the mongooses were held in the enclosure for ten minutes, which is much longer than they would need, to check all the cups. Some individuals may have simply been looking for a way out and solved the task by chance or continued checking cups without that intent. Third, we were measuring survival to adulthood, which is a relevant proxy for fitness, as less than half (41.8% of the pups in this dataset) lived to one year of age. While this is in line with the mortality rate of pups in the whole population. It may not accurately reflect lifetime reproductive success or ‘true fitness’ of surviving individuals. Furthermore, only a very small proportion of variance in pup survival (< 5%) was explained by escorts’ spatial memory performance, with environmental variation, predation etc. not controlled for here being important in driving patterns in mortality and other components of fitness^48,49^. Further research on whether the escort's spatial memory affects the pup’s spatial memory could determine whether spatial memory can be learned as seen in humans^50^, as well as testing other cognitive skills such as tool use^51^ to see whether this has a similar effect on young’s fitness. Another suggestion is looking at more "success" factors for the pups, such as fecundity or weight over time. Our study shows that the cognitive traits of carers in cooperatively breeding mammals may be a factor that influences young’s fitness and success.

## Data Availability

The datasets used in this study are available in the Fig Share repository, https://doi.org/10.6084/m9.figshare.23615046.v1.
